# Knowledge, Attitudes, and Perceptions Toward Organ Donation Among Patients Attending Primary Healthcare Centers: A Cross-Sectional Study

**DOI:** 10.7759/cureus.112652

**Published:** 2026-07-14

**Authors:** Abdullah M Al Qamshah, Faisal Alrasheed, Mohammed F Alserhani, Yasser Alkharashi, Ahmed Shalan S Alqarni, Nasser A Alqarni, Abdulaziz Braik S AlQarni, Khalid F Almuawi, Mohammed F Almuawi, Abdullah Aleisa

**Affiliations:** 1 Family Medicine, Ministry of National Guard Health Affairs, Riyadh, SAU; 2 Family Medicine, University of Bisha, Bisha, SAU; 3 Family Medicine, University Of Bisha, Bisha, SAU

**Keywords:** barriers to the organ donation, knowledge attitudes and practices, organ donation, organ donation awareness, organ transplant, primary healthcare

## Abstract

Background and objectives

Organ donation is a critical component of modern healthcare, yet the gap between the demand for organs and their availability remains substantial. This study evaluates knowledge, attitudes, and perceptions toward organ donation among patients at primary healthcare centers (PHCs).

Methods

A cross-sectional study was conducted among adult patients at PHCs from April 2025 to April 2026. A structured questionnaire assessed sociodemographic characteristics, knowledge, attitudes, previous exposure, and barriers to organ donation. Knowledge scores were classified as "good" (≥60%) or "poor" (<60%). Data were analyzed using SPSS version 28 (IBM Corp., Armonk, USA).

Results

A total of 330 participants were included, predominantly female (64.5%) and aged 18-25 years (37.9%). Most participants had prior awareness of organ donation (69.7%), while 26.1% had good overall knowledge. Liver (52.7%) and kidneys (51.2%) were the most recognized transplantable organs. Attitudes were moderately positive, with 52.7% supporting donation, while 41.5% were willing to donate after death. Barriers included fear of premature death (47.0%), bodily disfigurement (25.8%), and family influence (26.4%). Factors independently associated with good knowledge included younger age [≤25 years; odds ratios (OR)=3.25], university education (OR=6.15), student status (OR=2.85), single (OR=3.12), prior family discussions (OR=3.98), prior information exposure (OR=2.15), being a registered donor (OR=4.31), and personal or indirect experience with organ donation (OR=2.22).

Conclusion

Knowledge is insufficient despite moderately favorable attitudes. Family influence, religious uncertainty, and fear of disfigurement are key barriers. This study recommends targeted education, leveraging trusted providers, and incorporating religious guidance to improve awareness and promote organ donation.

## Introduction

Organ donation plays a key role in modern healthcare, offering a lifeline to individuals suffering from end-stage organ failure [1]. The ability to transplant vital organs is critical for medical science, providing patients with renewed life when faced with otherwise insurmountable health challenges [2]. Despite these advancements, there remains a clear gap between the demand for organs and the limited supply. The shortage leads to significant morbidity and mortality, as many patients remain on waiting lists with their lives in jeopardy [3]. Thus, there is an urgent need for strategies to increase public awareness and encourage donor participation, together with exploring innovative solutions to address this critical shortage [4]. Primary Healthcare Centers (PHCs) serve as the first point of contact for many individuals within the healthcare system, positioning them as key settings for shaping public knowledge and attitudes toward organ donation [5].

Globally, the mismatch between the number of available organs and the number of patients in need continues to be a major challenge [6]. WHO reports that only about 10% of the global need for organ transplants is met annually [7]. This shortfall is often worsened by limited awareness, cultural beliefs, and misconceptions surrounding organ donation [8]. In many communities, religious and ethical concerns, fear of medical procedures, and mistrust of the healthcare system contribute to low donation rates [9]. Addressing these barriers requires a thorough understanding of the knowledge and attitudes present in diverse populations. Evidence suggests that individuals with higher levels of knowledge are more likely to hold positive attitudes and express willingness to donate [10]. Nevertheless, misconceptions such as the belief that organ donation contradicts religious teachings or may compromise medical care persist even among educated populations [11].

Healthcare providers in PHCs can encourage organ donation when discussing it with patients and their families. Their own knowledge and attitudes are therefore critical, although research indicates that even healthcare professionals may have gaps in understanding or biases that limit their ability to advocate effectively for donation [12,13]. By focusing on patients attending PHCs, the study clarifies the importance of community-level engagement in assessing the organ shortage, aiming to improve health outcomes for individuals in need of life-saving transplants. Therefore, the study aims to assess the knowledge, attitudes, and perceptions toward organ donation among patients attending PHCs.

## Materials and methods

Design and setting

A cross-sectional study design was used to assess knowledge and attitudes toward organ donation among patients attending PHCs under the National Guard Health Affairs (NGHA) in Riyadh, Saudi Arabia, from April 2025 to April 2026.

Ethical approval

The study was conducted in accordance with the ethical principles outlined in the Declaration of Helsinki (2013), the International Ethical Guidelines for Health‑Related Research Involving Humans (CIOMS, 2016), and the World Health Organization Operational Guidelines for Ethics Committees that Review Biomedical Research. Ethical approval was obtained from the Research Advisory Committee and the Institutional Review Board of King Abdullah International Medical Research Center (KAIMRC) (Protocol No.: NRR25/014/4). All procedures were performed in compliance with the laws and regulations of the Kingdom of Saudi Arabia.

Population and sampling

The study population included all accessible and eligible adult patients (≥18 years) who could understand and respond to the questionnaire in Arabic or English and who provided informed consent. Patients under 18 years, those with cognitive impairments, NGHA healthcare workers, and participants with incomplete questionnaires were excluded. A multistage random sampling technique was used. In the first stage, PHCs under NGHA were stratified by geographic location, and a random selection of centers was made from each stratum. In the second stage, systematic random sampling was applied to select patients from daily patient lists, with every fifth eligible patient invited to participate. To facilitate wider reach and accessibility, the online questionnaires were distributed only to patients who had been identified through the sampling process at the selected PHCs and who had attended the centers during the study period. The survey link was shared directly with these selected individuals rather than disseminated publicly, thereby maintaining linkage to the sampled PHCs. The calculated minimum sample size was 250 participants and was adjusted to approximately 275 to account for an anticipated 10% non‑response rate, using the single‑proportion formula with a 95% confidence level and a 5% margin of error. An expected prevalence of 85% was assumed based on prior Saudi studies demonstrating a high baseline understanding of organ donation, including correct knowledge of the definition of organ donation (83.6%), knowledge of living kidney and liver donation (84.0% and 89.0%, respectively), and awareness of legal regulations governing organ donation (>85%) [14]. In the absence of a single reported composite knowledge score, this estimate was used solely for sample‑size determination. Recruitment continued until the end of the predefined data collection period, resulting in a final sample of 330 participants, which increased the study’s statistical precision without compromising the sampling strategy.

Data collection and instruments

Participants were recruited in person at the primary healthcare centers by trained research staff. After eligibility screening and informed consent, participants completed the questionnaire electronically using Google Forms, with the survey link shared directly with eligible participants on site via QR code, secure messaging including personal emails, WhatsApp, and/or SMS. Trained staff were available on site to provide clarification when requested, but responses were self‑administered to ensure privacy and reduce interviewer influence. No paper‑based or interviewer‑administered questionnaires were used. The data collection instrument was a structured questionnaire developed from an extensive literature review [5,8,9,11]. Content validity was assessed through review by subject‑matter experts in family and community medicine and public health, who evaluated the questionnaire for relevance, clarity, and comprehensiveness. The questionnaire was prepared in both Arabic and English. Forward translation and back‑translation procedures were used to ensure linguistic accuracy and conceptual equivalence between versions. A pilot study involving 15 participants was conducted to assess clarity, feasibility, and internal consistency. Feedback from the pilot resulted in minor wording refinements. The pilot participants were excluded from the final analysis. The reliability of the knowledge items was evaluated using Cronbach’s alpha, which was 0.71, indicating acceptable internal consistency.

The questionnaire included four main sections: (1) sociodemographic information, (2) knowledge about organ donation, (3) attitudes and perceptions toward organ donation, and (4) previous exposure to organ donation. Attitudes and perceptions toward organ donation were assessed using structured questions evaluating participants’ overall stance toward organ donation, willingness to donate after death, willingness for living donation, perception of organ donation as a noble act, and encouragement of family members to donate. Attitude items were measured using five‑point Likert scales ranging from strongly oppose (1) to strongly support/strongly agree (5), while willingness‑based items used ordered categorical responses. Perceived barriers to organ donation and previous exposure were assessed using multiple‑response categorical questions, including religious, cultural, familial, and informational factors. These variables were analyzed descriptively and examined for associations with knowledge outcomes.

Data analysis

All data were analyzed using SPSS version 28 (IBM Corp., Armonk, USA). Descriptive statistics, including frequencies and percentages, were used to summarize participants' sociodemographic characteristics, knowledge, attitudes, perceptions, previous exposure, and barriers regarding organ donation. The knowledge and awareness scoring was conducted as follows: each correct response in the knowledge section of the questionnaire was assigned 1 point, while incorrect or "I don't know" responses were assigned 0 points. The total score for each participant was then converted into a percentage of the maximum possible score. Categorization followed Bloom’s cut‑off points for knowledge, attitude and practice (KAP) studies [15]. For analysis, scores ≥60% were considered good knowledge. Associations between categorical variables, such as sociodemographic factors, prior exposure, and overall knowledge level, were assessed using Chi-square tests or Fisher's exact test where appropriate. To identify independent predictors of good knowledge, a multiple logistic regression analysis was performed, with results expressed as odds ratios (ORs) and 95% confidence intervals (CIs). A p-value <0.05 was considered statistically significant for all analyses.

## Results

The study included 330 participants, predominantly female (64.5%) and young adults aged 18-35 years (71.2%). Educational attainment was relatively high, with 48.8% having completed university education or higher, while 24.5% had secondary-level education. Students represented the largest occupational group (35.2%), followed by governmental employees (23.3%) and unemployed (17.9%), indicating a mixed academic and working population. Most respondents were unmarried (45.2%), with single individuals slightly outnumbering married participants (42.7%). The sample was largely characterized by low to moderate income, with over half reporting a monthly income below 5000 SR, suggesting limited financial resources among a substantial proportion of participants. Religiously, the population was highly homogeneous, with Muslims comprising 96.7% of respondents (Table 1).

**Table 1:** Socio-Demographic Characteristics of Patients Attending Primary Healthcare Centers in National Guard Health Affairs, Riyadh, Saudi Arabia

Socio-Demographics	No	%
Age in years		
18-25	125	37.9%
26-35	110	33.3%
36-45	22	6.7%
46-55	43	13.0%
>55	30	9.1%
Gender		
Male	117	35.5%
Female	213	64.5%
Educational level		
No formal education	28	8.5%
Basic education	60	18.2%
Secondary education	81	24.5%
University degree or higher	161	48.8%
Occupation		
Unemployed	59	17.9%
Student	116	35.2%
Governmental sector employee	77	23.3%
Private sector employee	48	14.5%
Retired	30	9.1%
Marital status		
Single	149	45.2%
Married	141	42.7%
Divorced / widowed	40	12.1%
Monthly income		
<5000 SR	183	55.5%
5000-10000 SR	99	30.0%
10001-15000 SR	33	10.0%
>15000 SR	15	4.5%
Religion		
Muslim	319	96.7%
Non-Muslim	11	3.3%

Overall awareness of organ donation was moderate, with 69.7% of participants reporting prior awareness of the concept. However, accurate understanding was limited, as only 46.7% correctly defined organ donation as the donation of organs or tissues for transplantation, while 31.5% were uncertain and others demonstrated misconceptions. Knowledge of transplantable organs was uneven; the liver (52.7%) and kidneys (51.2%) were the most frequently identified, whereas recognition of the heart (35.8%), lungs (20.6%), corneas (12.4%), and pancreas (10.0%) was substantially lower. Knowledge regarding the timing of organ donation was suboptimal, with only 39.7% correctly identifying that donation can occur after both brain and cardiac death, while 27.3% reported not knowing. Religious awareness was limited, as 47.6% believed organ donation is permitted in Islam, compared with 25.8% who believed it is not and 26.7% who were uncertain (Table 2). Awareness that living individuals can donate organs was reported by 55.8% of participants. Legal awareness was comparatively higher, with 68.5% recognizing that organ donation is legally regulated in Saudi Arabia. Despite this, overall knowledge remained poor, with 73.9% classified as having inadequate knowledge and awareness.

**Table 2:** Knowledge and Awareness Regarding Organ Donation among Patients Attending Primary Healthcare Centers in National Guard Health Affairs, Riyadh, Saudi Arabia

Knowledge and awareness	No	%
Have you heard about organ donation?		
Yes	230	69.7%
No	100	30.3%
Which of the following best describes organ donation?		
Donating organs or tissues for transplantation	154	46.7%
Giving blood for transfusion	22	6.7%
Participating in medical research	50	15.2%
I don’t know	104	31.5%
What organs can be donated?		
Liver	174	52.7%
Kidneys	169	51.2%
Heart	118	35.8%
Lungs	68	20.6%
Corneas	41	12.4%
Pancreas	33	10.0%
I don’t know	50	15.2%
When can organ donation occur?		
Both after brain death and cardiac death	131	39.7%
Only after cardiac death	73	22.1%
Only after brain death	36	10.9%
I don’t know	90	27.3%
Is organ donation permitted in Islam?		
Yes	157	47.6%
No	85	25.8%
I don’t know	88	26.7%
Can a living person donate an organ?		
Yes	184	55.8%
No	70	21.2%
I don’t know	76	23.0%
What is the legal status of organ donation in Saudi Arabia?		
Allowed and regulated by law	226	68.5%
Not allowed	43	13.0%
I don’t know	61	18.5%

Participants demonstrated overall moderately positive attitudes toward organ donation. A total of 33.3% supported and 19.4% strongly supported organ donation, while 30.3% expressed neutral attitudes; opposition was less common, reported by 7.9% and strong opposition by 9.1%. Willingness to donate organs after death was reported by 41.5% of participants, whereas 33.9% were unwilling and 24.5% were uncertain. Several factors influenced attitudes toward donation. Family approval was the most frequently reported determinant (26.4%), followed by fear of medical procedures (19.7%) and lack of trust in the healthcare system (18.8%). Altruistic motivations were reported by 15.5%, while religious beliefs influenced 14.5% of participants. Additional concerns included fear of body disfigurement (9.4%), financial considerations (6.7%), and inadequate communication or guidance (5.8%), with 36.7% indicating other unspecified factors. Perceptions of organ donation were generally favorable, with 23.6% agreeing and 24.5% strongly agreeing that it is a noble act, although 31.8% remained neutral. Regarding family influence, 40.3% would encourage family members to donate organs. Willingness toward living organ donation was more divided, with 24.5% willing and 13.9% very willing, while 36.9% expressed unwillingness, indicating greater hesitancy toward living donation compared to deceased donation (Table 3).

**Table 3:** Attitudes and Perceptions Toward Organ Donation among Patients Attending Primary Healthcare Centers in National Guard Health Affairs, Riyadh, Saudi Arabia

Attitude and perception	No	%
How do you feel about organ donation?		
Strongly oppose	30	9.1%
Oppose	26	7.9%
Neutral	100	30.3%
Support	110	33.3%
Strongly support	64	19.4%
Would you be willing to donate your organs after death?		
Yes	137	41.5%
No	112	33.9%
Not sure	81	24.5%
Factors influence your decision to donate organs		
Family approval	87	26.4%
Fear of medical procedures	65	19.7%
Lack of trust in the healthcare system	62	18.8%
Desire to help others	51	15.5%
Religious beliefs	48	14.5%
Fear of body disfigurement	31	9.4%
Financial concerns	22	6.7%
Lack of clear communication and guidance	19	5.8%
Others	121	36.7%
Do you believe organ donation is a noble act?		
Strongly disagree	48	14.5%
Disagree	18	5.5%
Neutral	105	31.8%
Agree	78	23.6%
Strongly agree	81	24.5%
Would you encourage your family members to donate organs?		
Yes	133	40.3%
No	98	29.7%
Not sure	99	30.0%
How willing are you to donate an organ while you are alive (living donation)?		
Very Unwilling	78	23.6%
Unwilling	44	13.3%
Neutral	81	24.5%
Willing	81	24.5%
Very Willing	46	13.9%

Previous exposure to organ donation was generally limited among participants. Only 42.4% reported having discussed organ donation with their family, whereas 57.6% had never engaged in such discussions. Regarding sources of information, media was the most commonly reported source (20.9%), followed by religious leaders (13.3%), healthcare providers (13.0%), and educational institutions (11.2%). Notably, a substantial proportion of participants (41.5%) reported not receiving any information from these sources. Concerning registration status, 33.3% reported being registered as organ donors, while 37.0% were not registered, and 29.7% indicated that they did not know how to register. Additionally, 59.4% of participants reported having personal or indirect exposure to organ donation, defined as either direct involvement as a donor or recipient or knowing someone who had been involved. This broad definition may have captured distant or indirect experiences rather than close personal involvement (Table 4).

**Table 4:** . Previous Exposure to Organ Donation among Patients Attending Primary Healthcare Centers in National Guard Health Affairs, Riyadh, Saudi Arabia

Previous Exposure to Organ Donation	No	%
Have you ever discussed organ donation with your family?		
Yes	140	42.4%
No	190	57.6%
Have you ever received information about organ donation from the following sources?		
Media (TV, radio, internet)	69	20.9%
Religious leaders	44	13.3%
Healthcare providers	43	13.0%
Educational institutions	37	11.2%
None of the above	137	41.5%
Are you registered as an organ donor?		
Yes	110	33.3%
No	122	37.0%
I don’t know how to register	98	29.7%
Have you or anyone you know ever been involved in organ donation (as a donor or recipient)?		
Yes	196	59.4%
No	134	40.6%

**Figure 1:**
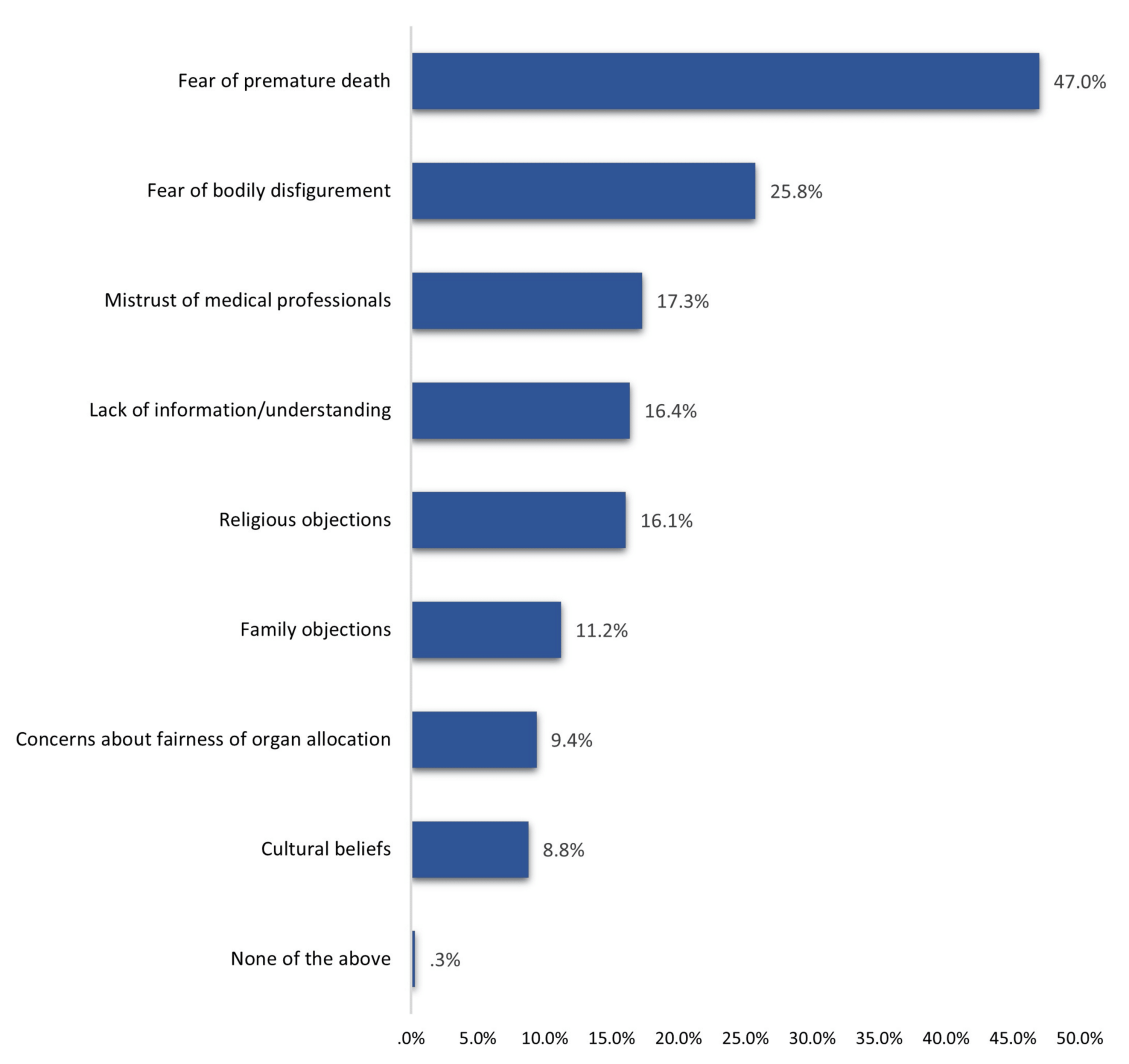
Reasons for Hesitation Toward Becoming an Organ Donor Among Patients Attending Primary Healthcare Centers

Religious and family-related concerns were the main reported barriers to organ donation. Religious uncertainty was common, with 41.2% expressing uncertainty and 21.2% unsure about religious permissibility; additionally, 16.7% believed the body should remain intact, and 12.4% reported discouragement from religious leaders, while only 8.5% had no religious objections. Family-related barriers were also prominent, as 46.7% were uncertain about family opposition, 22.4% feared negative family reactions, and 11.8% reported explicit family refusal, whereas 19.1% reported no family objections. Perceived availability of information was unclear, with 41.2% unsure, 31.5% believing information was sufficient, and 27.3% perceiving it as insufficient. Regarding informational needs, 37.3% required no additional information; among others, legal aspects (21.5%), donation procedures (13.3%), and religious guidance (13.0%) were the most requested (Table 5). Hesitation toward organ donation was primarily driven by fear‑related concerns. The most frequently reported reason was fear of premature death (47.0%), followed by fear of bodily disfigurement (25.8%). Other notable barriers included mistrust of medical professionals (17.3%) and lack of adequate information (16.4%). Religious objections (16.1%) and family opposition (11.2%) were also reported. Additional concerns involved perceived unfairness in organ allocation (9.4%) and cultural beliefs (8.8%). Only 0.3% of participants reported no concerns (Figure 1).

**Table 5:** Barriers and Informational Needs Regarding Organ Donation Among Patients Attending Primary Healthcare Centers in National Guard Health Affairs, Riyadh, Saudi Arabia

Barriers	No	%
If you have religious objections, which of the following best describes your concern?		
I believe my body should remain whole	55	16.7%
I am unsure of the religious permissibility	70	21.2%
My religious leader advises against it	41	12.4%
I have no religious objections.	28	8.5%
I don't know	136	41.2%
If you have family objections, which of the following best describes your concern?		
My family opposes it	39	11.8%
I am concerned about my family's reaction	74	22.4%
I have no family objections	63	19.1%
I don't know	154	46.7%
Do you think enough information about organ donation is available to the public?		
Yes	104	31.5%
No	90	27.3%
I don’t know	136	41.2%
What kind of information would you like to receive about organ donation?		
I do not need more information	123	37.3%
Legal aspects	71	21.5%
The process of donation	44	13.3%
Religious perspectives	43	13.0%
Success rates of transplants	27	8.2%
Financial incentives or compensation	22	6.7%

Overall knowledge was significantly associated with several socio‑demographic and exposure factors. Younger participants (18-25 years) showed the highest proportion of good knowledge (49.6%), with good knowledge declining sharply with age to 6.7% among those >55 years (p=0.001). Educational level was strongly associated, as 44.7% of participants with a university degree demonstrated good knowledge compared with ≤13.6% among those with secondary education or less (p=0.001). Students had the highest level of good knowledge (50.0%), whereas retirees (6.7%) and governmental employees (11.7%) demonstrated lower levels (p=0.001). Singles showed higher good knowledge (47.0%) than married (10.6%) or divorced/widowed participants (2.5%) (p=0.001). Gender showed no significant association. Prior information exposure appeared to be associated with higher knowledge in some contexts; participants who had discussed organ donation with family (42.1%), were registered donors (50.9%), or had personal or indirect experience with donation (32.1%) (all p≤0.002) showed relatively higher levels of good knowledge. However, knowledge levels varied across information sources, and a notable proportion of participants reporting no specific information source also demonstrated good knowledge, indicating that information exposure alone does not fully explain knowledge differences (Table 6).

**Table 6:** Factors Associated With the Overall Knowledge and Awareness of Organ Donation P: Pearson X^2^ test, ^: Exact Probability test, * p<0.05 (significant)

Factors	Overall knowledge and awareness level				p-value
	Poor		Good		
	No	%	No	%	
Age in years					.001*^
18-25	63	50.4%	62	49.6%	
26-35	94	85.5%	16	14.5%	
36-45	20	90.9%	2	9.1%	
46-55	39	90.7%	4	9.3%	
>55	28	93.3%	2	6.7%	
Gender					.692
Male	85	72.6%	32	27.4%	
Female	159	74.6%	54	25.4%	
Educational level					.001*
No formal education	27	96.4%	1	3.6%	
Basic education	58	96.7%	2	3.3%	
Secondary education	70	86.4%	11	13.6%	
University degree or higher	89	55.3%	72	44.7%	
Occupation					.001*
Unemployed	50	84.7%	9	15.3%	
Student	58	50.0%	58	50.0%	
Governmental sector employee	68	88.3%	9	11.7%	
Private sector employee	40	83.3%	8	16.7%	
Retired	28	93.3%	2	6.7%	
Marital status					.001*
Single	79	53.0%	70	47.0%	
Married	126	89.4%	15	10.6%	
Divorced / widow	39	97.5%	1	2.5%	
Religion					.045*^
Muslim	233	73.0%	86	27.0%	
Non-Muslim	11	100.0%	0	0.0%	
Have you ever discussed organ donation with your family?					.001*
Yes	81	57.9%	59	42.1%	
No	163	85.8%	27	14.2%	
Have you ever received information about organ donation from the following sources?					.001*
Healthcare providers	32	74.4%	11	25.6%	
Media (TV, radio, internet)	56	81.2%	13	18.8%	
Religious leaders	41	93.2%	3	6.8%	
Educational institutions	29	78.4%	8	21.6%	
None of the above	86	62.8%	51	37.2%	
Are you registered as an organ donor?					.001*
Yes	54	49.1%	56	50.9%	
No	101	82.8%	21	17.2%	
I don’t know how to register	89	90.8%	9	9.2%	
Have you or anyone you know ever been involved in organ donation (as a donor or recipient)?					.002*
Yes	133	67.9%	63	32.1%	
No	111	82.8%	23	17.2%	

Multivariable logistic regression identified several independent predictors of good knowledge. Participants aged ≤25 years were more likely to have good knowledge (OR=3.25; p=0.001). Higher education was the strongest predictor, with university‑educated participants showing markedly higher knowledge (OR=6.15; p=0.001). Students (OR=2.85; p=0.001) and single participants (OR=3.12; p=0.001) were significantly more knowledgeable than other groups. Prior exposure was influential: discussing organ donation within the family (OR=3.98; p=0.001), receiving information from any source (OR=2.15; p=0.008), being a registered donor (OR=4.31; p=0.001), and having personal or indirect donation experience (OR=2.22; p=0.004) were all associated with higher knowledge. Notably, in bivariate analysis, religion showed a statistically significant association with knowledge levels; however, this association did not remain significant in the multivariable logistic regression model after adjustment for other covariates. Similarly, gender was not independently associated with knowledge in the multivariable analysis (Table 7).

**Table 7:** Multiple Logistic Regression Analysis of Factors Associated with Good Knowledge About Organ Donation Among Patients Attending PHCs in NGHA AOR: adjusted odds ratio, CI: confidence interval, * p<0.05 (significant)

Predictor	AOR	95% CI	p-value
Age ≤25 vs >25 years	3.25	1.85 – 5.70	0.001*
Gender (Male vs Female)	0.91	0.55 – 1.52	0.712
Education (University vs ≤Secondary)	6.15	3.25 – 11.63	0.001*
Occupation (Student vs Others)	2.85	1.60 – 5.07	0.001*
Marital status (Single vs Married/Divorced)	3.12	1.77 – 5.50	0.001*
Religion (Muslim vs non-Muslim)	2.11	0.95 – 4.66	0.065
Family discussion (Yes vs No)	3.98	2.28 – 6.95	0.001*
Received info (Yes vs No)	2.15	1.22 – 3.78	0.008*
Registered donor (Yes vs No/I don’t know)	4.31	2.39 – 7.76	0.001*
Personal/known experience (Yes vs No)	2.22	1.30 – 3.80	0.004*

## Discussion

This study provides a comprehensive assessment of knowledge, attitudes, and perceptions toward organ donation among 330 patients attending PHCS. Our findings demonstrate a significant knowledge gap alongside moderately favorable attitudes, underscoring the need for targeted educational interventions in primary healthcare settings.

Knowledge of organ donation

The findings demonstrate that although more than two‑thirds of participants had heard about organ donation, only about one‑quarter demonstrated good overall knowledge, indicating a marked gap between awareness and understanding. This finding is consistent with Alobaidi et al., who showed widespread basic awareness but persistent deficiencies regarding key issues such as brain death and donor registration [14]. Similar patterns have been reported regionally, with studies from Oman and Tunisia demonstrating generally favorable awareness but uneven depth of knowledge across domains [16,17]. In contrast, higher correct identification of organ donation has been reported in Qatar (62.7%) and Jordan (>90%), highlighting variability in knowledge levels across countries [18,19].

More than one‑third of participants correctly identified that organ donation can occur after both brain and cardiac death, indicating persistent knowledge gaps. Limited understanding of brain death has been widely reported in Gulf countries, with a recent survey showing that 87.1% of citizens lack full awareness of the meaning of brain death, with 89.5% unaware of the medical and legal standards for donation eligibility [20]. This reflects a prevailing perception of death as cessation of heartbeat and breathing. In this study, the liver and kidneys were the most frequently recognized transplantable organs, consistent with their disease burden and media visibility. Awareness declined substantially for other organs, with only about one‑third identifying the heart and fewer recognizing the lungs, corneas, or pancreas as transplantable.

Regarding religious views, only 47.6% believed that organ donation is permitted in Islam, while 26.7% were uncertain, indicating substantial religious ambiguity. This aligns with prior evidence identifying religious uncertainty as a major barrier to organ donation among Muslim populations [21]. In contrast, legal awareness was higher, as 68.5% of participants correctly recognized that organ donation is legally permitted and regulated in Saudi Arabia, reflecting broad awareness of the national regulatory framework [22].

Attitudes and perceptions

Nearly half of the participants expressed positive attitudes toward organ donation, with 33.3% supporting and 19.4% strongly supporting it; however, only 41.5% reported willingness to donate their own organs after death, highlighting a gap between approval and personal commitment. Similarly, Alobaidi et al. found that indecision regarding organ donor registration predominated across all sociodemographic groups, with a substantial proportion of participants holding favorable attitudes yet remaining undecided about actual registration [14]. Our findings concur with Al-Abdulghani et al., who found that family approval was the most influential factor affecting willingness, followed by fear of medical procedures and distrust in the healthcare system [23]. Despite Islamic teachings that emphasize saving lives, altruistic motives such as helping others were reported by relatively few participants. Nonetheless, perceptions remained largely favorable, as 48.1% viewed organ donation as a noble act and 40.3% indicated they would encourage family members to donate [24]. Our findings on willingness toward living organ donation indicated mixed preferences rather than a clear preference over deceased donation, which contrasts with the preference for living over deceased donation reported in many Muslim‑majority countries [24-26].

Barriers to organ donation

Our study identified multiple barriers to organ donation at individual, family, and systemic levels. Concerns related to fear of premature death and bodily disfigurement emerged as prominent barriers to organ donation, reflecting underlying cultural beliefs regarding bodily integrity after death. Similar concerns have been reported in previous studies, including those by Al‑Abdulghani et al. [21], suggesting that such perceptions remain persistent across comparable populations. Religious considerations also played a role; however, uncertainty regarding the permissibility of organ donation appeared more influential than explicit religious opposition. This finding aligns with earlier literature indicating that inadequate religious guidance, rather than doctrinal prohibition, often contributes to hesitancy toward organ donation. Similarly, Kattoah et al. identified the inclusion of religious authorities to address theological concerns as a priority for improving donation rates [20]. Family-related barriers were substantial, as over one‑fifth expressed concern about their family's reaction, consistent with the requirement for family consent within the Saudi transplantation system. These findings were in line with internationally reported family concerns [27,28], in Islamic countries including Saudi Arabia [29,30]. Informational barriers were also evident, with a large proportion reporting no prior exposure to information through media, religious leaders, healthcare providers, or educational institutions.

Factors associated with knowledge

Multivariable analysis showed that higher educational attainment was strongly associated with good knowledge, with university graduates having more than six times higher odds of good knowledge compared with participants with secondary education or less [10]. Younger participants (≤25 years), students, and single individuals also demonstrated significantly higher knowledge levels, indicating gaps among older adults and those with lower education. Prior exposure was important: participants who had received information or discussed organ donation with their family were nearly four times more likely to have good knowledge, suggesting mutual reinforcement between knowledge and family dialogue. Gender was not significantly associated with knowledge after adjustment, despite previous reports of gender differences. However, Alobaidi et al. found that females reported higher willingness to register than males, with a significant association (χ²=10.3, p=0.006) [14]. Religion was not independently associated with knowledge in the multivariable model [30]. Although all non‑Muslim participants demonstrated poor knowledge, this finding should be interpreted cautiously given the small number of non‑Muslim participants in the sample and may reflect contextual or informational factors rather than a true religious difference.

Strengths and limitations

This study has several strengths, including its focus on primary healthcare patients, a population that has received limited attention in previous research. Face‑to‑face recruitment at PHCs, complemented by electronic questionnaire distribution, improved accessibility and response rates while allowing clarification of questions and minimizing missing data. Nonetheless, several limitations should be mentioned. The multistage random sampling approach was intended to improve representativeness within the NGHA PHCs; however, participants were recruited in person with all questionnaire’s self‑administered electronically. This approach may have introduced selection bias related to digital literacy and access, potentially favoring younger or more technologically proficient participants. The study was conducted exclusively within NGHA PHCs in Riyadh, which may limit generalizability to other healthcare sectors or regions of Saudi Arabia. Social desirability bias may have influenced responses, mainly regarding willingness to donate and registration status. Finally, the exclusion of patients with incomplete questionnaires, while methodologically sound, may have introduced selection bias if those with missing data differed systematically from completers.

## Conclusions

The findings of the current study revealed that among patients attending PHCS, knowledge about organ donation is inadequate despite moderately favorable attitudes. The discrepancy between general support and personal willingness, coupled with high levels of uncertainty regarding religious permissibility and family reactions, represents a critical barrier to increasing organ donation rates. Educational interventions in primary healthcare settings, delivered through trusted providers and incorporating religious guidance, are recommended and may help improve awareness and willingness toward organ donation.
